# Transcription Factors of the Alx Family: Evolutionarily Conserved Regulators of Deuterostome Skeletogenesis

**DOI:** 10.3389/fgene.2020.569314

**Published:** 2020-11-23

**Authors:** Jian Ming Khor, Charles A. Ettensohn

**Affiliations:** Department of Biological Sciences, Carnegie Mellon University, Pittsburgh, PA, United States

**Keywords:** Alx transcription factors, skeletogenesis, chondrogenesis, osteogenesis, deuterostome evolution, neural crest cell, biomineralization, calcification

## Abstract

Members of the *alx* gene family encode transcription factors that contain a highly conserved Paired-class, DNA-binding homeodomain, and a C-terminal OAR/Aristaless domain. Phylogenetic and comparative genomic studies have revealed complex patterns of *alx* gene duplications during deuterostome evolution. Remarkably, *alx* genes have been implicated in skeletogenesis in both echinoderms and vertebrates. In this review, we provide an overview of current knowledge concerning *alx* genes in deuterostomes. We highlight their evolutionarily conserved role in skeletogenesis and draw parallels and distinctions between the skeletogenic gene regulatory circuitries of diverse groups within the superphylum.

## Introduction

Biomineralization, the formation of mineral by living organisms, is an exceptionally widespread phenomenon and is thought to have evolved independently and rapidly in many different metazoan phyla through the deployment of a wide range of biomineralization mechanisms and chemistries. Depending on the type and extent of the mineral components, biomineralized tissues are used for structural support, resource acquisition, and protection. There are three predominant classes of biogenic mineral in metazoans: calcium carbonates, calcium phosphates, and silica. The carbonate and phosphate salts of calcium are widely used as skeletal material by vertebrates and invertebrates, while silica biomineralization is prevalent in sponges (Wang et al., 2010b). The emergence of biomineralization during the Cambrian Explosion, followed by evolutionary modifications of these biomineralization programs, gave rise to the diverse biomineralized structures found in modern metazoans (Knoll, 2003; Zhuravlev and Wood, 2018).

Within the deuterostome superphylum, only vertebrates and echinoderms produce extensive biomineralized skeletal structures. The vertebrate endoskeleton consists primarily of the skull, vertebrae, ribs, and limb bones all of which are composed of matrix proteins (e.g., collagens) and calcium phosphate crystals. Vertebrate biomineralization is predominantly orchestrated by chondrogenic cells (chondrocytes) and osteogenic cells (osteoblasts and osteoclasts). The vertebrate skeleton is formed during early development by cartilage and/or connective tissue membranes, which are subsequently replaced by bony tissues through the process of ossification. There are two forms of ossification, endochondral and intramembranous ossification. Endochondral ossification is associated with the formation of long bones and requires the presence of a hyaline cartilage template formed by chondrocytes (Mackie et al., 2008). During vertebrate embryonic development, chondrocytes are derived from neural crest cells, somitic mesodermal cells, and lateral plate mesodermal cells (see review by Hirasawa and Kuratani, 2015). Developmental cues signal the cartilage matrix to calcify. This prevents the diffusion of nutrients into the matrix and results in chondrocyte apoptosis, allowing blood vessels to invade the cartilage cavities. Osteoblasts, derived from common osteochondroprogenitor or directly from chondrocytes (Yang et al., 2014), and osteoclasts, derived from erythron-myeloid progenitors (Jacome-Galarza et al., 2019), then transform the calcified cartilage into biomineralized bone (Mackie et al., 2008). During intramembranous ossification, spongy bones are formed when osteoblasts directly deposit biomineral on extracellular sheets of mesenchymal connective tissues (Percival and Richtsmeier, 2013). This process is commonly involved in the formation of flat bones found in the skull, mandible, and clavicles. Whether intramembranous or endochondral ossification arose first during vertebrate evolution remains unclear (Cervantes-Diaz et al., 2017; Wood and Nakamura, 2018; Brazeau et al., 2020).

All adult echinoderms produce calcite-based endoskeletons that consist of the test, teeth, and spines. In most species, the adult form arises from a swimming, feeding larva *via* metamorphosis, and these two life history stages bear little morphological resemblance to one another. In some echinoderm clades, specifically echinoids (sea urchins) and ophiuroids (brittle stars), the feeding larva also possesses an intricate and extensive calcitic endoskeleton, which is first laid down during embryonic development and further elaborated after feeding begins. The founder cells of the embryonic skeletogenic lineage, the large micromeres, arise early in development and are specified by a combination of localized maternal factors and unequal cell division. At the mesenchyme blastula stage, the large micromere descendants undergo an epithelial-to-mesenchyme transition (EMT) and ingress into the blastocoel as primary mesenchyme cells, or PMCs (see reviews by Ettensohn, 2020; McClay et al., 2020). After ingression, PMCs extend filopodia and migrate along the blastocoel wall, gradually adopting a ring-like configuration near the equator of the embryo. As the PMCs migrate, their filopodia fuse, forming a cable-like cytoplasmic strand that connects the cells in a syncytial network. Amorphous calcium carbonate and associated proteins are then secreted into an intercellular space within the cytoplasmic cable, where the biomineral matures and grows, eventually producing the elaborate, branched skeletal elements (spicules) of the larva (Wilt, 2002; McIntyre et al., 2014; Shashikant et al., 2018).

Due to differences in mechanisms underlying axial patterning, developmental timing, and embryological structures, it is often difficult to deduce morphological homology. Although the biomineralized tissues found in different metazoan phyla are not considered homologous in the strictest sense, recent comparative studies have revealed common elements across different biomineralization systems. This has led to the recognition of a possible “biomineralization toolkit;” an ancestral gene regulatory network (GRN) consisting of signaling and gene regulatory pathways that was independently co-opted and fine-tuned for biomineralization in diverse animal taxa. One common regulator of deuterostome skeletogenesis is the Alx transcription factor family, which has been shown to have an ancient, conserved role in this process in both vertebrates and echinoderms. In this review, we examine the current state of knowledge concerning deuterostome *alx* genes, with a focus on their role in skeletogenesis.

## Phylogenetic Distribution of *ALX* Genes in Deuterostomes

**Figure 1:**
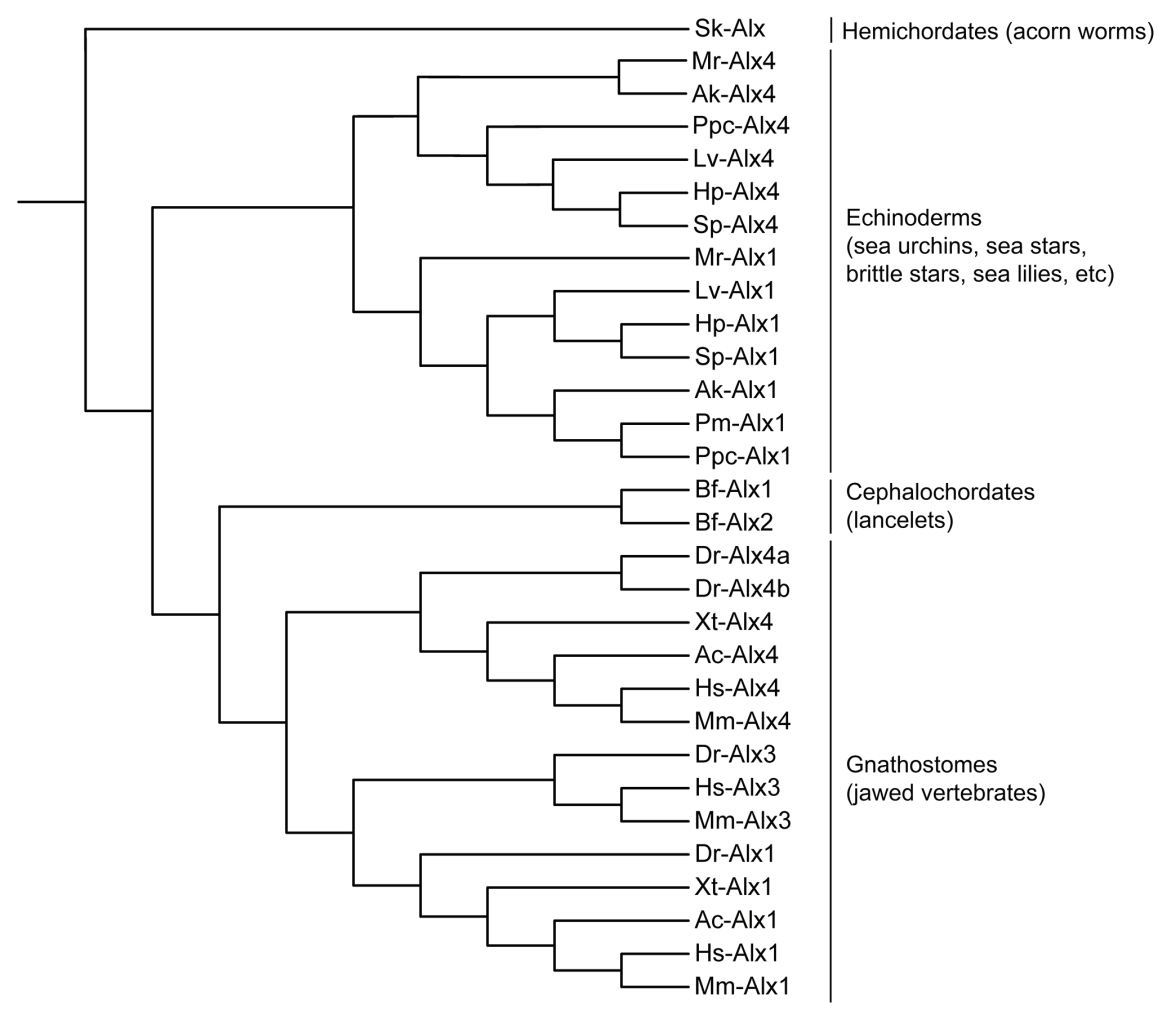
Molecular phylogeny of Alx proteins (adapted from McGonnell et al., 2011; Koga et al., 2016). Branch lengths are arbitrary. Sk, *Saccoglossus kowalevskii* (acorn worm); Lv, *Lytechinus variegatus* (euechinoid sea urchin); Hp, *Hemicentrotus pulcherrimus* (euechinoid sea urchin); Sp, *Strongylocentrotus purpuratus* (euechinoid sea urchin); Mr, *Metacrinus rotundus* (sea lily); Ak, *Amphipholis kochii* (brittle star); Pm, *Patiria miniata* (sea star); Ppc, *Patiria pectinifera* (sea star); Bf, *Branchiostoma floridae* (lancelet); Dr, *Danio rerio* (zebrafish); Xt, *Xenopus tropicalis* (frog); Ac, *Anolis carolinensis* (lizard); Hs, *Homo sapiens* (human); Mm, *Mus musculus* (mouse).

The *alx* gene family encodes Paired-class homeodomain transcription factors that contain a highly conserved DNA-binding homeodomain and a C-terminal Otp, Aristaless, and Rax (OAR) domain, features that are shared by many Paired-class homeodomain proteins. Phylogenetic and comparative genomic studies have revealed considerable variability in the number of *alx* genes in different deuterostomes, pointing to a complex evolutionary pattern of lineage-specific gene duplication and loss (Figure 1; adapted from McGonnell et al., 2011; Koga et al., 2016). Hemichordates possess a single *alx* gene (Koga et al., 2016) while echinoderms have two (*alx1* and *alx4*; Ettensohn et al., 2003; Koga et al., 2016). In contrast, humans and mammals possess three *alx* genes (*alx1*/*cart1*, *alx3*, and *alx4*) that arose through two duplication events. Through the course of evolution, one of the paralogues, *alx3*, was lost from amphibian and reptile lineages (McGonnell et al., 2011). Additionally, ray-finned fishes such as zebrafish acquired two paralogues of *alx4*, designated *alx4a* and *alx4b*, as a result of a separate, whole genome duplication event (McGonnell et al., 2011). The lancelets have two *alx* genes. In *Branchiostoma floridae*, these two genes (*Bf-alx1* and *Bf-alx2*) are located close to each other in the genome and have very similar intron-exon organizations. Molecular phylogenetic analysis of Alx proteins indicate that Bf-Alx1 and Bf-Alx2 form a monophyletic group, providing further support for the view that they arose from a lineage-specific gene duplication event (Figure 1; Koga et al., 2016).

## Developmental Expression and Function of *ALX* Genes in Jawed Vertebrates

Members of the *alx* gene family are expressed in several mesenchymal tissues during the embryogenesis of jawed vertebrates (gnathostomes), a group that includes most of the vertebrate species used for developmental studies. These genes are expressed most prominently in distinct but partially overlapping patterns in neural crest-derived craniofacial mesenchyme and in mesenchyme of the limb bud, both of which are sources of cartilage and bone (Zhao et al., 1994; Qu et al., 1997a; ten Berge et al., 1998; Beverdam and Meijlink, 2001). Other sites of embryonic expression have also been reported, including the head mesoderm, sclerotome of the somite (another tissue that produces cartilage and bone), hair follicles, dental papillae of teeth, and parts of the developing urogenital system (Zhao et al., 1994; Hudson et al., 1998; ten Berge et al., 1998; Bothe et al., 2011; Wang et al., 2019).

In the developing head, genes of the *alx* family are expressed by neural crest cells, which give rise to cartilages and bones of the skull, jaw, and middle ear, as well as other derivatives (see reviews by Santagati and Rijli, 2003; Noden and Trainor, 2005). Consistent with this pattern of expression, perturbations of *alx* genes commonly result in severe craniofacial malformations, including frontonasal dysplasia and the reduction or malformation of many neural crest-derived skeletal elements (Table 1). In mice, loss-of-function mutations of *alx1*/*cart1* or *alx4* also lead to other cranial abnormalities such as anencephaly and lacrimal gland aplasia (Zhao et al., 1996; Garg et al., 2017), although these effects are likely to be secondary consequences of defects in neural crest cells, which provide essential signals that regulate the development of the brain and eye (Zhao et al., 1996; Bhattacherjee et al., 2009; Le Douarin, 2012; Garg et al., 2017). While *alx3*-null mice appear normal, *alx3*/*alx4* double mutant mice exhibit severe frontonasal dysplasia and cranial skeletal defects that are more extreme than those observed in *alx4* mutant mice, revealing non-equivalent but overlapping functions of these highly similar proteins (Beverdam et al., 2001).

**Table 1:** Summary of expression patterns, mutations, perturbations, and diseases associated with *alx* genes across different deuterostome phyla.

Organism	Gene	Expression Pattern	Reference	Mutation/Perturbation	Disease/Mutational Effect	Reference
Human	*alx1*	n.d.	n.d.	Whole-gene deletion and homozygous homeodomain splice-site mutation (c.531+1G>A)	Frontonasal dysplasia, characterized by microphthalmia and severe facial clefting	Uz et al., 2010
				Reciprocal translocation t(1;12)(p32.1;q21.3) resulting in enhanced gene expression	Microcephaly, language impairment, and mental retardation	Liao et al., 2011
	*alx3*	n.d.	n.d.	Nonsense (c.543T>A; p.Y191X), frameshift (c.578_581delCTGA; p.T193RfsX137), and splice-site (c.595-2A>T) mutations within homeodomain	Frontonasal dysplasia (frontorhiny)	Twigg et al., 2009
				Nonsense mutation within homeodomain (c.604C>T; p.Q202X), resulting in premature stop	Frontonasal dysplasia (frontorhiny)	Ullah et al., 2018
	*alx4*	n.d.	n.d.	Deletion and insertion mutation (c.1080_1089delGACCCGGTGCinsCTAAGATCTCAACAGAGATGGCAACT; p.D326fsX21), resulting in frameshift and loss of OAR domain	Mild frontonasal dysplasia and enlarge parietal foramina	Bertola et al., 2013
				Deletions (c.385_394del, c.417_418del), point mutation (c.620C>A), and duplication (c.456_465dup)	Enlarged parietal foramina	Mavrogiannis et al., 2006
				Deletion (c.504delT; p.D169X), resulting in premature stop and loss of homeodomain; point mutation in homeodomain (c.815G>C; p.R272P)	Enlarged parietal foramina	Wuyts et al., 2000
				Nonsense mutation (c.793C>T; p.R265X)	Frontonasal dysplasia	Kayserili et al., 2009
				Point mutation (c.653G>A; p.R218Q) in homeodomain nuclear localization signal	Enlarged parietal foramina	Valente et al., 2004
				Deletion (c.291delG; p.Q98SfsX83) resulting in frameshift and premature stop	Frontonasal dysplasia	El-Ruby et al., 2018
				Point mutations (c.19G_T; p.V7F, c.631A>G; p.K211E, c.917C>T; p.P306L)	Nonsyndromic craniosynostosis	Yagnik et al., 2012
Mouse	*alx1*	Craniofacial region (frontonasal head mesenchyme), lateral plate mesoderm, and limb bud mesenchyme	Beverdam and Meijlink, 2001; Zhao et al., 1994	Homozygous null mutant	Acrania and anencephaly	Zhao et al., 1996
	*alx3* and *alx4*	Overlapping expression in the craniofacial region (frontonasal head mesenchyme), lateral plate mesoderm, and limb bud mesenchyme. *alx3* is expressed in parts of the developing urogenital system. *alx4* is expressed in hair follicles and dental papillae of teeth.	Qu et al., 1997a; Hudson et al., 1998; ten Berge et al., 1998	Homozygous double *alx3*/*alx4* mutant	Frontonasal dysplasia and preaxial polydactyly	Beverdam et al., 2001
Zebrafish	*alx1*, *alx3*, *alx4a*, and *alx4b*	Overlapping expression in the frontonasal mesenchyme, periocular mesenchyme, mandible arch, and the prospective palate. *alx1* is expressed in the head mesoderm.	Dee et al., 2013; Wang et al., 2019	Knockdown using *alx1* antisense morpholino oligonucleotide	Defective neural crest migration and craniofacial malformations	Dee et al., 2013
				Knockdown using *alx3* antisense morpholino oligonucleotide	No significant effect	Dee et al., 2013
Cattle	*alx4*	n.d.	n.d.	Duplication (c.714_734dupTCACCGAGGCCCGCGTGCAG) within the homeodomain	Tibial hemimelia syndrome	Brenig et al., 2015
Cat	*alx1*	n.d.	n.d.	In frame deletion of homeodomain sequences (c.496_507delCTCTCAGGACTG)	Frontonasal dysplasia	Lyons et al., 2016
Frog	*alx1* and *alx4*	Frontal mesenchyme near the eyes	McGonnell et al., 2011	n.d.	n.d.	n.d.
Chicken	*alx1* and *alx4*	Craniofacial region (frontonasal head mesenchyme)	Bothe et al., 2011; McGonnell et al., 2011	n.d.	n.d.	n.d.
Lamprey	*alx*	Trabecular cartilaginous elements near the eye, upper lip mesenchyme and parts of the branchial basket cartilage	Cattell et al., 2011; Kuratani et al., 2016; Square et al., 2017	n.d.	n.d.	n.d.
Lancelet	*alx*	Paraxial mesoderm, pharyngeal arch mesoderm, and gut diverticulum	Meulemans and Bronner-Fraser, 2007	n.d.	n.d.	n.d.
Thin-spined sea urchin	*alx1*	Primary mesenchyme cells in embryos and juvenile skeletogenic centers in late stage larvae	Ettensohn et al., 2003;	Knockdown using *alx1* antisense morpholino oligonucleotide	Loss of skeletogenic cell specification	Ettensohn et al., 2003
			Gao and Davidson, 2008	Overexpression of Alx1 via mRNA microinjection into fertilized eggs	Ectopic activation of the skeletogenic program in mesodermal lineage cells	Ettensohn et al., 2003
	*alx4*	Primary mesenchyme cells and coelomic mesoderm in embryos	Rafiq et al., 2012; Koga et al., 2016	n.d.	n.d.	n.d.
Pencil urchin	*alx1*	Skeletogenic mesenchyme lineage cells	Erkenbrack and Davidson, 2015	Knockdown using *alx1* antisense morpholino oligonucleotide	Loss of skeletogenic cell specification	Erkenbrack and Davidson, 2015
Sea star	*alx1*	Juvenile skeletogenic centers in late stage larvae	Gao and Davidson, 2008	Overexpression of Alx1 via mRNA microinjection into fertilized eggs	Upregulation of sea star orthologues of sea urchin skeletogenic genes during embryogenesis	Koga et al., 2016
Sea cucumber	*alx1*	Skeletogenic mesenchyme lineage cells	McCauley et al., 2012	Knockdown using *alx1* antisense morpholino oligonucleotide	Loss of skeletogenic cell specification	McCauley et al., 2012
Brittle star	*alx1*	Skeletogenic mesenchyme lineage cells and adult skeletogenic centers in juveniles	Czarkwiani et al., 2013; Koga et al., 2016	n.d.	n.d.	n.d.
Acorn worm	*alx*	Coelomic mesoderm	Koga et al., 2016	n.d.	n.d.	n.d.

n.d., not determined.

During early zebrafish development, the expression of *alx1* alone is detected in migrating neural crest cells, while at later stages, *alx1*, *alx3*, *alx4a*, and *alx4b* exhibit overlapping patterns of expression in the craniofacial mesenchyme (Dee et al., 2013; Wang et al., 2019). *Alx1* is also transiently expressed in the cranial paraxial mesoderm at early developmental stages (Wang et al., 2019). Perturbation of Alx1 expression using antisense morpholino oligonucleotides (MOs) produces severe craniofacial defects in zebrafish, similar to results seen in the mouse, inhibition of *alx3* alone results in no significant craniofacial abnormalities (Dee et al., 2013). In developing frog and chick embryos, both *alx1* and *alx4* are expressed robustly in the craniofacial mesenchyme (Bothe and Dietrich, 2006; McGonnell et al., 2011; Square et al., 2015).

Genes of the *alx* family are also expressed in the mesodermal compartment of the limb buds. At early embryonic stages, these genes are expressed specifically in an anterior, proximal zone while later in development they are also expressed at the distal margin (Qu et al., 1997b). The anterior, proximal zone of expression may include sites where skeletal elements of the shoulder and pelvic girdles (the scapula and pelvis, respectively) form, although this has not been shown directly. The skeletal elements of the limb girdles have complex embryological origins that are only partially understood. The scapula may arise from three sources: somatic mesoderm of the lateral plate, somite-derived dermamyotome, and neural crest, while the pelvis likely arises from somatic mesoderm and sclerotome (Young et al., 2019). Genetic knockouts in mice have revealed essential and partially redundant roles for *alx1*, *alx3*, and *alx4* in the formation of the superior/anterior portion of the scapula blade (and in the development of the clavicle) and have shown that *alx1* expression in this region is under the direct control of the transcription factors Emx2 and Pbx1 (Kuijper et al., 2005a,b; Capellini et al., 2010). Similarly, compound *alx1*:*alx4* and *alx3*:*alx4* double mutants reveal overlapping roles for these genes in the formation of the pelvic skeleton (Kuijper et al., 2005b; Young et al., 2019). Unlike the neural crest-derived skeleton of the head, the scapula and pelvis both form by endochondral ossification, and defects are observed in both the cartilaginous and bony compartments of these skeletal elements when the function of *alx* family genes is compromised.

A striking developmental consequence of alx4 null mutations is preaxial polydactyly – the formation of one or more supernumerary anterior digits (Forsthoefel, 1963; Qu et al., 1997b). This effect is associated with the formation of an ectopic, anterior zone of polarizing activity (ZPA) in the limb bud and concomitant, anterior expression of *sonic hedgehog* (*shh*; Chan et al., 1995; Qu et al., 1997a,b; Takahashi et al., 1998). At relatively late developmental stages, Shh signaling is required for polydactyly to develop in *alx4*-null mutants, but it has been proposed that *alx4* also plays an earlier, Shh-independent role in anterior-posterior patterning (Kuijper et al., 2005b). The expression domains of *alx4* and *shh* during limp outgrowth are established, in part, by mutual repression (Kuijper et al., 2005b; Matsubara et al., 2017).

Consistent with the results of experimental gene perturbations, genetic association studies in several vertebrate species have shown that polymorphisms in *alx* genes are associated with phenotypic variations in skeletal development. A genome-wide scan of genetic diversity between two closely related species of Darwin’s finches has revealed that polymorphism within the *alx1* gene is strongly associated with beak morphology (Lamichhaney et al., 2015). Linkage analysis and genome-wide association studies have also identified a small 12 bp deletion in the *alx1* gene that is associated with frontonasal dysplasia in Burmese cats (Lyons et al., 2016). Furthermore, variations in the number of repeats in the coding region of *alx4* are quantitatively associated with polydactyly in the Great Pyrenees dog breed (Fondon and Garner, 2004), and a 20 bp duplication in the *alx4* gene is linked to congenital tibial hemimelia (loss or shortening of the tibia) in Gallow cattle (Brenig et al., 2015). Taken together, these findings suggest that an ancient *alx* gene may have constituted a conserved, core element of the ancestral vertebrate skeletogenic GRN and that gene duplication followed by divergence of the paralogs with respect to their developmental expression and/or biochemical properties has produced multiple *alx* family members with overlapping functions.

Considered as a whole, these studies show that members of the vertebrate *alx* gene family play a conserved, prominent role in the development of the cranial and appendicular skeletons. In contrast, they do not appear to mediate the development of the sclerotome-derived, axial skeleton of the trunk (the vertebrae and ribs). Members of the *alx* gene family may also have other, less well-characterized, developmental functions, although some of the effects of mutations in these genes on non-skeletal tissues are likely to be indirect. In the cranial region, it is well-established that *alx*-family genes are expressed robustly and selectively by neural crest cells (Rice et al., 2003; Dee et al., 2013; Garg et al., 2017), a cell population that gives rise to both cartilage and membranous bone. Expression of *alx* family genes is not uniform in all regions of the developing head, however, and it has been hypothesized that this contributes to a regulatory code that controls the region-specific identity of the cranial neural crest (Square et al., 2017). With respect to appendage development, the expression of *alx*-related genes is associated with skeleton-forming potential of mesenchymal cell that will form proximal elements of the limb girdles (clavicle, scapula, and pelvis; Young et al., 2019). The embryological origins and the precise developmental fates of these cells, as well as that of other cells of the developing limb that express *alx*-related genes, are not well-characterized.

## Developmental Expression of *ALX* Genes in Other Chordates

In basally-derived (jawless) vertebrates and cephalochordates (amphioxus), animals that possess only cartilaginous skeletons, *alx*-family genes are expressed in patterns consistent with a role in skeletogenesis. The single lamprey *alx* gene is expressed at high levels in the trabecular cartilaginous elements near the eye, in a region that may be derived from mesoderm or from the cranial neural crest (Kuratani et al., 2016; Square et al., 2017). Cephalochordates have stiff, acellular pharyngeal endoskeletons that contain fibrillar collagen, and the adult form has a cartilaginous oral skeleton that supports the cirri (Jandzik et al., 2015). Amphioxus lacks a neural crest, and the embryonic cell lineage that produces the oral skeleton has not been identified. One study has examined the expression of *alx*-related genes in cephalochordates and reported expression in the somites and right gut diverticulum at neurula/early larval stages (Meulemans and Bronner-Fraser, 2007). At present, the function of *alx*-related genes in jawless vertebrates and amphioxus has not been explored through gene perturbation studies.

## Developmental Expression and Function of *ALX* Genes in Echinoderms

In echinoderm clades that form larval skeletons, *alx1* is one of the earliest regulatory genes expressed during development, and it plays a pivotal role in specifying the fate of PMCs, the embryonic skeletogenic cells (Ettensohn et al., 2003; Erkenbrack and Davidson, 2015; Dylus et al., 2016; Shashikant et al., 2018). Transcription of *alx1* can be detected as early as the 56-cell stage specifically in the large micromeres (the progenitors of PMCs), and expression remains restricted to this cell lineage throughout embryogenesis (Ettensohn et al., 2003). Perturbation of Alx1 expression using MOs inhibits PMC specification while overexpression of Alx1 results in ectopic activation of the skeletogenic program in other mesodermal lineages. Furthermore, experimental ablation of PMCs leads to the activation of *alx1* and downstream components of the skeletogenic GRN by non-skeletogenic mesoderm (NSM) cells, which ultimately reform a larval skeleton (Ettensohn et al., 2007). The ectopic activation of *alx1* is essential for NSM cells to acquire skeletogenic properties, although this activation occurs by a mechanism distinct from that which normally operates in the large micromeres (Oliveri et al., 2008; Sharma and Ettensohn, 2011; Ettensohn and Adomako-Ankomah, 2019). Remarkably, the removal of NSM cells *via* microsurgical removal of the archenteron as well as PMCs results in the activation of *alx1* and formation of a skeleton by presumptive endoderm cells (Sharma and Ettensohn, 2011).

**Figure 2:**
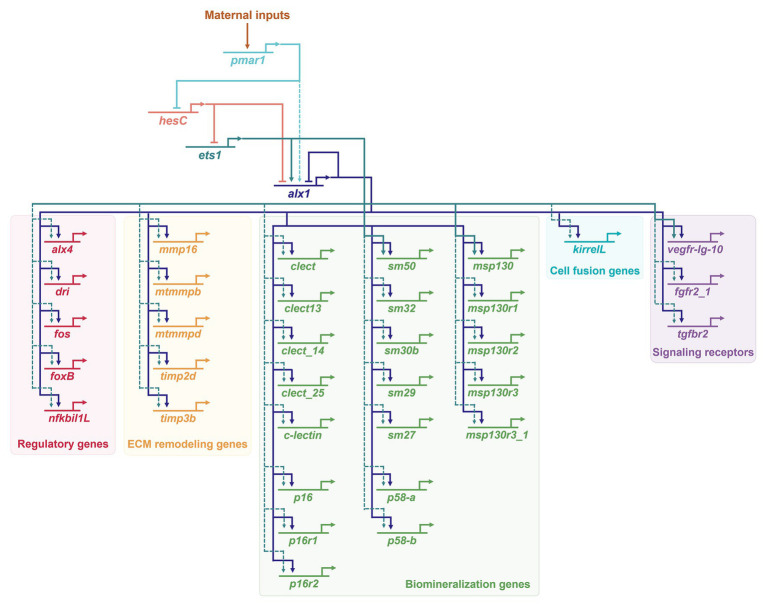
Activation of Alx1 in euechinoids (*S. purpuratus*) and regulatory inputs into primary mesenchyme cell (PMC) effector genes. Only a small number of more than 420 effector genes differentially expressed in PMCs (Rafiq et al., 2014) is shown here. A large subset of effector genes receives regulatory inputs from both Ets1 and Alx1 (Rafiq et al., 2014). Positive regulatory inputs by Ets1 and Alx1 into *msp130*, *sm50*, and *vegf-Ig-10* are described in (Oliveri et al., 2008). Direct targets of the sea urchin Alx1 (Khor et al., 2019) define a genetic subcircuit that impinges on almost all aspect of PMC morphogenesis, including directional cell migration, extracellular matrix (ECM) remodeling, cell-cell fusion, and biomineralization. Dashed arrows indicate interactions that may be indirect. For additional information regarding the developmental functions of the specific effector genes shown here, see Shashikant et al. (2018) and references therein.

The role of *alx1* in the skeletogenic GRN in euechinoid sea urchins has been especially well-characterized (Figure 2). Alx1 provides positive inputs into almost half of the ~420 genes that are differentially expressed by PMCs, highlighting the pivotal role of Alx1 in establishing skeletogenic cell identity (Rafiq et al., 2014). A recent chromatin immunoprecipitation sequencing (ChIP-seq) study determined that many of these genes, including both regulatory (i.e., transcription factor-encoding) and effector (i.e., differentiation) genes, are direct targets of *alx1* (Khor et al., 2019). A second transcription factor, Ets1, collaborates with Alx1 in the co-regulation of a large fraction of genes differentially expressed by PMCs (Rafiq et al., 2014), in many cases through a feed-forward mechanism (i.e., Ets1 > Alx1, Ets + Alx1 > effector gene; Yamasu and Wilt, 1999; Amore and Davidson, 2006; Oliveri et al., 2008; Yajima et al., 2010; Shashikant et al., 2018; Khor et al., 2019). Downstream effector genes that are regulated by Alx1 include those that directly mediate biomineralization (e.g., those that encode secreted spicule matrix proteins that are incorporated into the biomineral) and those that mediate skeletogenesis through signaling pathways and morphogenetic cell behaviors (Figure 2).

The *alx1* gene is also expressed specifically in skeletogenic cells of cidaroids (pencil urchins) and holothuroids (sea cucumbers), and is required and for skeletogenesis in these species (McCauley et al., 2012; Erkenbrack and Davidson, 2015). The *alx1* gene is robustly expressed in adult skeletogenic centers, even in sea stars, which lack a larval skeleton (Gao and Davidson, 2008; Czarkwiani et al., 2013; Gao et al., 2015). Comparative studies have revealed many similarities in the gene regulatory programs of skeletogenic cells in the larva and adult (Richardson et al., 1989; Gao and Davidson, 2008; Killian et al., 2010; Czarkwiani et al., 2013; Gao et al., 2015). Hence, it is widely thought that the larval skeleton arose within the echinoderms by co-option of an adult skeletogenic program. Moreover, ectopic expression of sea urchin or sea star *alx1* in sea star embryos is sufficient to activate several sea star orthologs of sea urchin skeletogenic genes (Koga et al., 2016). These findings confirm the critical role that Alx1 plays in establishing skeletogenic identity across all echinoderms at all life history stages, supporting the view that this function was present in the last common ancestor of echinoderms.

Echinoderms also possess a paralog of *alx1*, known as *alx4*. The two genes are directly adjacent to one another in the sea urchin genome, suggesting that they arose through gene duplication. The sister group to echinoderms, the hemichordates, possess a single *alx* gene, suggesting that the gene duplication occurred after the divergence of echinoderms from hemichordates (Koga et al., 2016). The *alx4* gene, like *alx1*, is expressed by skeletogenic PMCs, but *alx4* is also expressed by presumptive coelomic pouch cells at the tip of the archenteron (Rafiq et al., 2012; Koga et al., 2016). The function of *alx4* has not been experimentally determined but it has been proposed to be involved in coelom development as the single *alx* gene in hemichordates is expressed in the coelomic mesoderm. As adult hemichordates possess only small biomineralized elements (Cameron and Bishop, 2012), these observations suggest that *alx1* gained enhanced skeletogenic function in echinoderms secondarily. Structure-function analysis of Alx1 and Alx4 in euechinoid sea urchins has revealed that the gene duplication event permitted the functional specialization of Alx1 through changes in intron-exon organization and the acquisition of a novel protein motif known as the D2 domain (Khor and Ettensohn, 2017). As noted above, a recent genome-wide ChIP-seq study showed that a large part of the embryonic skeletogenic GRN of sea urchins is directly regulated by Alx1, including many morphoeffector genes that are also expressed in adult skeletogenic centers. Hence, a heterochronic shift in *alx1* expression from adult skeletogenic centers to the embryonic skeletogenic cells may have been sufficient to co-opt a substantial subcircuit of biomineralization genes and ultimately transfer skeletogenesis into the embryo (Khor et al., 2019).

## A Suite of Deuterostome Biomineralization Effector Genes Regulated by *ALX1* in Echinoderms

Studies on vertebrates and echinoderms have identified many examples of closely related genes that mediate biomineralization in both taxa, such as collagens and carbonic anhydrases (see reviews by Veis, 2011; Le Roy et al., 2014). Here, we focus on effector genes that have been identified as direct targets of Alx1 in echinoderms (sea urchins) and that have vertebrate counterparts implicated in chondrogenesis or osteogenesis. Though much is known about the interactions between regulatory genes and signaling pathways in vertebrate neural crest and chondrogenic GRNs (Cole, 2011; Simoes-Costa and Bronner, 2015), direct transcriptional inputs into biomineralization genes that are the downstream effectors of these networks have not been elucidated. Such information will be crucial to definitively assess homology between echinoderm and vertebrate skeletogenic GRNs.

## VEGF and Vegfr

One of the direct targets of sea urchin *alx1* in biomineralizing cells is the vascular endothelial growth factor (VEGF) receptor, *vegfr-Ig-10*, one of the two *vegfr* genes in sea urchins (Duloquin et al., 2007; Rafiq et al., 2014; Khor et al., 2019). During embryonic development, *vegfr-Ig-10* expression is restricted to PMCs, while its ligand, *vegf3* is expressed in the ectoderm specifically in the regions that lie adjacent to two ventro-lateral clusters of PMCs that initiate biomineral formation. MO-based knockdown of Vegf3 or Vegfr-Ig-10 results in the downregulation of skeletogenic genes and lack of embryonic skeleton formation, while ectopic expression of Vegf3 results in supernumerary skeletal elements and irregular branching (Duloquin et al., 2007; Adomako-Ankomah and Ettensohn, 2013). The *vegfr-Ig-10* gene is also expressed in adult skeletogenic centers, even in clades that lack a larval skeleton (Gao and Davidson, 2008; Morino et al., 2012). Other comparative studies in echinoderms have found a strict correlation between the expression of *vegf3*/*vegfr-Ig-10* and the formation of an embryonic skeleton (Duloquin et al., 2007; Morino et al., 2012; Adomako-Ankomah and Ettensohn, 2013; Erkenbrack and Petsios, 2017; Erkenbrack and Thompson, 2019). Remarkably, human VEGFA is able to rescue skeleton formation in sea urchin embryos that lack endogenous Vegf3 expression (Morgulis et al., 2019).

During vertebrate endochondral ossification, the cartilage intermediate is replaced by bone in a process that is partly regulated by the formation of a vascular network (see review by Green et al., 2015). Chondrocytes stimulate vasculogenesis through the secretion of VEGF ligands (Carlevaro et al., 2000). *In vitro* studies show that VEGF ligands (VEGFA, VEGFB, and VEGFC) and VEGF receptors (VEGFR2 and VEGFR3) are expressed by chondrocytes and chondrogenic cells, and autocrine signaling through this pathway regulates morphogenesis and differentiation (Carlevaro et al., 2000; Bluteau et al., 2007). Inhibition of *Vegf* signaling perturbs ossification and bone elongation by promoting chondrocyte proliferation rather than osteoblast differentiation (Gerber et al., 1999; Jacobsen et al., 2008). Mice with conditional deletion of *vegfa* in skeletal lineage cells exhibit thinner bones and decreased skeletal mineralization (Duan et al., 2015). Moreover, conditional deletion of *vegfr2* results in reduced osteogenic differentiation (Duan et al., 2015).

## MMPs and TIMPs

Another class of effector protein common to echinoderm and vertebrate biomineralization consists of matrix remodeling proteins such as matrix metalloproteases (MMPs) and tissue inhibitors of metalloproteinases (TIMPs). MMPs constitute a class of enzymes that function in the degradation of extracellular matrix (ECM) proteins (see review by Rose and Kooyman, 2016). In sea urchins, chemical inhibition of MMPs reversibly blocks spiculogenesis by PMCs *in vivo* and *in vitro* (Roe et al., 1989; Ingersoll and Wilt, 1998). In vertebrates, *mmp-13* (collagenase-3) is expressed specifically in chondrocytes (Tuckermann et al., 2000). Additionally, *in vitro* studies have shown that silencing of *mmp-2* by siRNA disrupts chondrogenic differentiation of mesenchymal stem cells while treatment with a MMP-2 activator stimulates chondrogenesis (Jin et al., 2007). TIMPs have been reported to be the primary endogenous inhibitors of MMPs and are involved in regulating the function of MMPs in many systems (Brew and Nagase, 2010). Overexpression of *timp-3* in mice induces defects in skeletal development and growth (Poulet et al., 2016). In contrast, knockdown of *timp-1* results in upregulated proliferation of mesenchymal stem cells while delaying osteogenic differentiation (Liang et al., 2019).

## SLC26

Many members of the solute carrier (SLC) family of membrane transport proteins are differentially expressed in the PMCs (Rafiq et al., 2014; Barsi et al., 2015). In addition, Alx1 directly regulates the expression of several members of the SLC5 and SLC26 sub-families, including *Slc26a5/1* and *Slc5a11/2* (Rafiq et al., 2014; Khor et al., 2019). While there are data pointing to SLCs that are essential for echinoderm skeletogenesis, mainly *Slc4a10* (Hu et al., 2018) and *Slc26a2/7* (Piacentino et al., 2016), the functions of the proteins that are directly regulated by Alx1 have not been tested. In vertebrates, *Slc26a2*, a sulfate transporter, has been shown to be highly expressed in developing and mature cartilage (Haila et al., 2001). Mice homozygous for mutations in *Slc26a2* exhibit chondrodysplasia, a condition characterized by growth defects and skeletal dysplasia due to reduced chondrocyte proliferation (Forlino et al., 2005). Similarly, mutations in human *Slc26a2* also results in chondrodysplasia (Superti-Furga et al., 1996; Jackson et al., 2012).

## Fam20c

One of the direct targets of sea urchin Alx1 is *fam20C*, which encodes a kinase of the FAM20 (family with sequence similarity 20) family (Rafiq et al., 2014; Khor et al., 2019). In vertebrates, members of this family are highly expressed in mineralized tissues, such as teeth and bone (Hao et al., 2007; Wang et al., 2010a). Fam20C is a secreted kinase responsible for the phosphorylation of secreted proteins, many of which are known to be involved in biomineralization (Tagliabracci et al., 2012). Mutations in the human *fam20C* gene cause Raine syndrome, an autosomal recessive disorder characterized by defects in bone development, including microcephaly, cleft palate, and osteosclerosis (Simpson et al., 2007; Rafaelsen et al., 2013; Takeyari et al., 2014; Seidahmed et al., 2015). *In vitro* mutational analyses suggest that Fam20C is involved in the differentiation and mineralization of mouse mesenchymal cells (Hao et al., 2007; Liu et al., 2017), and *fam20C*-null mice exhibit severe biomineralization defects, such as lesions in bones and teeth (Vogel et al., 2012; Wang et al., 2012; Du et al., 2015).

## Otopetrin

Sea urchin Alx1 also provides positive inputs directly into *otop2L*, the single sea urchin ortholog of the vertebrate *otopetrin* genes (Rafiq et al., 2014; Khor et al., 2019). Otopetrins are multi-pass transmembrane proteins that function as proton channels (Saotome et al., 2019). In vertebrates, these proteins play an essential role in regulating the timing, size, and shape of the developing otoconia, extracellular calcium carbonate biominerals that are required for vestibular functions (Hughes et al., 2004; Sollner et al., 2004; Kim et al., 2010). During mouse and zebrafish embryogenesis, *otop1* is highly expressed in the developing sensory epithelium of the ear (Hurle et al., 2003; Hughes et al., 2004). In zebrafish, MO-based knockdown of Otop1 results in otolith malformations (Hughes et al., 2004; Sollner et al., 2004). Moreover, *otop1* knockout mice also lack otoconia, a phenotype that has been attributed to mis-regulation of intracellular calcium levels (Hughes et al., 2007; Kim et al., 2010). The function of the echinoderm Otop2L protein has not been examined.

## *ALX* Genes and the Evolution of Deuterostome Biomineralization

Among present-day deuterostomes, extensive biomineralized skeletons are found only in echinoderms and vertebrates. It is inherently difficult to reconstruct the underlying evolutionary relationships between the skeletogenic programs of these two groups, which diverged >600 million years ago (Peterson and Eernisse, 2016). It is widely accepted that the ancestral chordate possessed only a cartilaginous skeleton (Rychel et al., 2006; Murdock and Donoghue, 2011; Jandzik et al., 2015; Keating et al., 2018), strongly supporting the view that biomineralized skeletons appeared independently in vertebrates and echinoderms, and therefore, are not homologous in the strictest sense. This does not, of course, resolve the question of whether common embryological and/or genetic mechanisms were deployed to create a biomineralized skeleton in these two groups; i.e., whether skeletogenesis in the two clades is an example of “deep homology” (Shubin et al., 2009). The presence of collagenous pharyngeal cartilage in both cephalochordates and hemichordates supports the view that this was an ancestral feature of deuterostomes that was later lost in echinoderms (Rychel and Swalla, 2007; Jandzik et al., 2015). Moreover, a recent analysis of chondrogenesis in protostomes (horseshoe crabs and cuttlefish) suggests that a more ancient, SoxE and collagen-based chondrogenic gene network was present in the last common ancestor of all Bilateria (Tarazona et al., 2016), providing further support for the view that echinoderm ancestors at one time also possessed cartilage-forming cells. It should be noted that although there is no evidence for definitive cartilage in modern echinoderms, there are mesoderm-derived populations of mesenchymal cells that produce connective tissue containing fibrillar collagen (Suzuki et al., 1997; Whittaker et al., 2006; Goh and Holmes, 2017).

The evolutionary relationships among the skeletogenic cell lineages of vertebrates that express *alx*-related genes and the *alx1*-expressing cells of echinoderms are uncertain. With respect to echinoderms, considerable evidence supports the view that *alx1* arose very early in echinoderm evolution through gene duplication, relatively quickly acquired a robust, biomineralization-related function, and was subsequently co-opted into the early embryo in echinoderm taxa that possess larval skeletons (echinoids and ophiuroids; Khor and Ettensohn, 2017; Shashikant et al., 2018). The biomineralizing cells of the ancestral echinoderm, which were likely of mesodermal origins, expressed *alx1*, *ets1*, *erg*, *vegfr*, and other components of a core skeletogenic program, as well as an assortment of more rapidly evolving biomineralization effector proteins (Gao and Davidson, 2008; Dylus et al., 2018; Erkenbrack and Thompson, 2019; Li et al., 2020). To draw inferences concerning the evolution of *alx* gene expression and function more deeply within Ambulacraria (echinoderms and hemichordates), it will be important to learn more about the single *alx* gene of hemichordates, including its pattern of expression, gene targets, and role in the formation of the small, calcareous skeletal elements of adult hemichordates (Cameron and Bishop, 2012) and to more precisely determine the embryological origins of the *alx1*-expressing cells of adult echinoderms, which are more relevant to the ancestral echinoderm condition than the more commonly studied larval forms.

In vertebrates, as noted above, the embryonic lineages of cells in the limbs and limb girdles that express *alx1*-related genes have not been mapped precisely, although many of these cells are presumably derived from the somatic layer of the lateral plate mesoderm, a major source of limb skeletal tissue. There is evidence that chondrocytes and osteoblasts of the limb are derived from a common, mesenchymal precursor cell and that the specialization of these two cell types depends upon regulatory functions of *sox9* (a member of a small number of paralogous, *soxE*-family genes in vertebrates) and other sox genes in the chondrogenic lineage, and *runx2* and *osterix* in the osteoblast lineage (Akiyama et al., 2005; Cervantes-Diaz et al., 2017; Lefebvre, 2019; Marín-Llera et al., 2019). Because *alx*-related genes have not been linked directly to the regulatory network that underlies limb skeletogenesis, and because Sox and Runx proteins are not currently known to be associated with skeleton formation in echinoderms, there is presently no obvious similarity between the GRN circuitry that controls skeletal development in the vertebrate limb and the echinoderm skeleton. As noted above, during limb girdle (scapula) development, *alx1* is co-regulated by Emx2 and Pbx1, but the orthologous echinoderm genes have not been studied in detail.

Perhaps the best-characterized cell population in vertebrates that employs *alx*-related genes in biomineralization is the cranial neural crest. There is agreement that a definitive neural crest is found only in vertebrates, but the evolutionary history of this cell population, particularly the origins of the skeletogenic (cranial) compartment, remains a subject of much debate (Jandzik et al., 2015; Rothstein et al., 2018; Cheung et al., 2019; York and McCauley, 2020). Like the program of skeletogenesis in the limb, the formation of cranial neural crest-derived cartilage and bone is believed to progress through the specification of a common osteochondral progenitor, with important contributions by Sox9 and Runx2 in chondrocyte and osteoblast differentiation, respectively (Martik and Bronner, 2017; Dash and Trainor, 2020). The regulatory inputs into *alx*-family genes in the cranial neural crest are unknown, however, and only one direct target (fgf10) has been identified (Garg et al., 2017). Thus, the precise role of *alx*-related genes in the dynamic differentiation program of skeletogenic cranial neural crest cells and their connections to the underlying gene regulatory circuitry remain to be elucidated.

As noted above, in jawless vertebrates and cephalochordates, the expression patterns of *alx*-family genes are consistent with a possible function in the formation of the cartilaginous, pharyngeal skeletons of these animals. A detailed comparison of the expression patterns of *alx*-family genes in lampreys and jawed vertebrates has led to the hypothesis that an expansion of the domain of *alx*-expressing cells may have supported the expansion of the cranial skeleton during vertebrate evolution (Square et al., 2017). With the important caveat that expression data are sparse in these taxa and function studies are lacking, these observations are consistent with the hypothesis that *alx*-related genes were expressed (at least) in the anterior, pharyngeal mesoderm of ancient chordates, in cells that produced pharyngeal cartilage (Kaucka and Adameyko, 2019).

A hypothesis that emerges from these comparative studies is that a rudimentary, ancestral program of chondrogenesis, perhaps deployed in mesenchyme cells derived from embryonic mesoderm, was present in the ancestral deuterostome and provided a suitable gene regulatory system onto which biomineralization-promoting circuitry could be layered. We propose that in echinoderms, gene duplication was followed by the neo-functionalization of *alx1*; i.e., the acquisition of a new role in robustly mediating biomineralization, as reflected by the direct transcriptional inputs this transcription factor provides into a large fraction of biomineralization effector genes (Rafiq et al., 2014; Khor et al., 2019). A similar (and presumably independent) neo-functionalization may have occurred in vertebrates, but the transcriptional targets of vertebrate *alx*-family genes have not been characterized, and therefore, it is not known whether they include effectors of biomineralization. It should be noted that possible signals of evolutionary conservation between echinoderms and vertebrates in this context would likely be obscured by the well-documented, rapid evolution of many biomineralization-related proteins (Kawasaki et al., 2004; Livingston et al., 2006; Marin et al., 2016; McDougall and Degnan, 2018). Presumably, the independent duplication of *alx*-family genes in echinoderms and vertebrates initially involved the sharing and/or duplication of *cis*-regulatory elements among paralogs, as indicated by the overlapping patterns of expression of paralogous *alx*-family genes in both taxa. The recruitment of duplicated, *alx-*related genes to a biomineralization-related function would likely have been facilitated if the ancestral gene was already expressed in an embryonic tissue that produced an extensive extracellular matrix, a prerequisite for the assembly and growth of biomineral (Bolean et al., 2017; Murshed, 2018). In this regard, it will be valuable to characterize more completely in representative deuterostomes the cell lineages that express *alx*-family genes and to better reconstruct the evolutionary relationships among those cell lineages.

## Author Contributions

JK and CE contributed to the conception of the review and co-wrote the paper. Both the authors contributed to the article and approved the submitted version.

### Conflict of Interest

The authors declare that the research was conducted in the absence of any commercial or financial relationships that could be construed as a potential conflict of interest.
